# Blepharoptosis and corneal epithelial thickness alterations, is there any relation?

**DOI:** 10.1186/s12886-024-03556-w

**Published:** 2024-07-12

**Authors:** Seyed Mohsen Rafizadeh, Seyed Ali Sonbolestan, Haniyeh Zeidabadinejad, Mohammad-Ali Abtahi

**Affiliations:** 1 Department of oculo-facial plastic and reconstructive Surgery, Eye Research Center, Farabi Hospital, Tehran University of Medical Sciences, Tehran, Iran; 2 Department of cornea and anterior segment, Farabi Hospital, Tehran University of Medical Sciences, Tehran, Iran; 3 Farabi Hospital, Eye Research Center, Tehran University of Medical Sciences, Tehran, Iran

**Keywords:** Corneal epithelial thickness, Blepharoptosis

## Abstract

**Background:**

To compare the epithelial thickness map of ptotic eyes of blepharoptosis patients with contralateral non- ptotic eyes.

**Methods:**

Unilateral blepharoptosis patients were enrolled consecutively. Patients were underwent full ophthalmologic examination and their demographic data such as age and gender and specific ptosis findings e.g. the cause and duration, MRD-1, and levator palpebralis superioris function were registered. Anterior segment imaging for epithelial thickness measurements was done using the Avanti RTVue-XR platform. The corneal epithelial thickness maps of ptotic and non-ptotic eyes were compared.

**Results:**

44 patients with unilateral blepharoptosis were included in the study. 27 (61.4%) of them were female and 17 (38.6%) cases were male. The mean of the patients’ ages was 24.40 ± 15.16 years. Ptotic eyes had significantly thinner superior (*p* = 0.000), superior-temporal (*p* = 0.000) and superior-nasal (*p* = 0.005) sectors of the cornea and slightly thicker corneal epithelium (CE) in the inferior-nasal sector. The correlation of difference of superior-inferior CE was evaluated with different parameters including patient’s age (*p* = 0.457), type of blepharoptosis (*p* = 0.786), duration of blepharoptosis (*p* = 0.477) and MRD1 (*p* = 0.248), but no correlation was found.

**Conclusions:**

This study revealed that lid position in blepharoptosis may have effects on the corneal epithelial thickness map. Because of the lower position of upper eyelid, a thinning effect on superior corneal sectors may happen.

## Background

The outermost layer of the cornea is the corneal epithelium (CE), which has a constant turnover and relatively stable thickness in normal eyes. This layer in constant relationship to the pre-corneal tear film, provides defensive and optical properties vital to maintaining a healthy ocular surface [1]. In contrast to this relative stability in normal eyes, epithelium has the great ability to alter in different conditions to mask the changes of the underlying stroma with great plasticity [2, 3].

Several previous studies, have evaluated the central epithelial thickness (CET) with either very high-frequency ultrasound (VHF-US) or spectral-domain optical coherence tomography (SD-OCT) in different normal or abnormal eye conditions. These modalities can produce maps from a large diameter of the cornea [4].

According to these studies several differences in the epithelial thickness map (ETM) were found in relation to eyelid position or eyelid abnormalities. With rare exceptions [5], almost all studies found thicker CET in the inferior cornea than in the superior cornea of normal eyes [6–20]. Some authors related this difference to mechanical rubbing of the upper eyelid to superior corneal epithelium, possibly thinning the superior ET [18, 21, 22].

Total CET profiles may show higher variability in older age groups [12, 23, 24], particularly in the superior parts of the cornea [25] especially in the paracentral and midperipheral [26, 27] or limbal areas [26].

Studies on ETM in patients with allergic conjunctivitis showed a significant decrease in average CET of paracentral and midperipheral annuli [28].On the other hand, comparison of ETM in patients with mild congenital myogenic ptosis to normal eyes showed significantly thinner ET and higher ETM SD possibly due to the thinning of the superior ET in all sectors in the ptosis group compared to inferior sectors [29].

So, in this study we aimed to compare the epithelial thickness map of ptotic eyes of blepharoptosis patients with contralateral non- ptotic eyes in a contralateral eye study.

## Methods

This cross-sectional study was conducted in oculoplastic and cornea departments, Farabi Eye Hospital, Tehran University of Medical Sciences, between September 2022 and July 2023.

Unilateral blepharoptosis patients were enrolled consecutively. Blepharoptosis was defined as an asymmetric marginal reflex distance 1 (MRD-1, corneal reflex distance to the upper eyelid margin) in which the affected upper lid is 1.5 mm or more lower than the normal side.

Patients were underwent full ophthalmologic examination and their demographic data such as age and gender and specific ptosis findings e.g. the cause and duration, MRD-1, and levator palpebrae superioris (LPS) function (the distance (in mm) of excursion of the upper eyelid margin from far downgaze to up gaze while the frontalis muscle is held immobile with the examiner’s hand) were registered.

Exclusion criteria included age (less than 5 years and more than 75 years), presence of other corneal pathologies, any history of previous ocular surface or corneal surgeries, use of any topical drops, ocular surface disorders like dry eye, stem cell deficiency, clinical keratoconus, thyroid eye disease, continuous contact lens use in the last month and any history of refractive surgeries including post-photorefractive keratotomy (post-PRK), post-laser-assisted in situ keratomileusis (post-LASIK), post-small incision lenticule extraction (post-SMILE) or post-intracorneal ring segment (post- ICRS).

Anterior segment imaging for epithelial thickness measurements was done using the Avanti RTVue-XR platform (Optovue, Fremont, CA, USA) system which is a Fourier-domain OCT system. The OCT system has a working wavelength of 830 nm and operates at a scan speed of 26,000 axial scans per second. It has a depth resolution of 5 μm (full-width–half-maximum) in tissue [30]. An add-on lens of the corneal adaptor module was also be used. All of the enrolled patients were trained not to use any topical medications two hours earlier than imaging. All of the scans were performed by one trained technician with no delay longer than five minutes between the two eyes to decrease any bias. Imaging was done between 11 AM and 1 PM for all subjects to avoid confounding diurnal variation of measurements. The imaging room temperature was kept at 23–25 °C with a relative humidity of 40–45% [31]. Although newer measurement protocols evaluate the corneal pachymetry in 9 mm scans, due to the lack of this type of evaluation, in this study conventional “Pachymetry + Cpwr” scan pattern (6.0 mm scan diameter, 8 radials, 1024 axial scans each, repeated 5 times) centered at the pupil center was used to map the cornea.

**Fig. 1:**
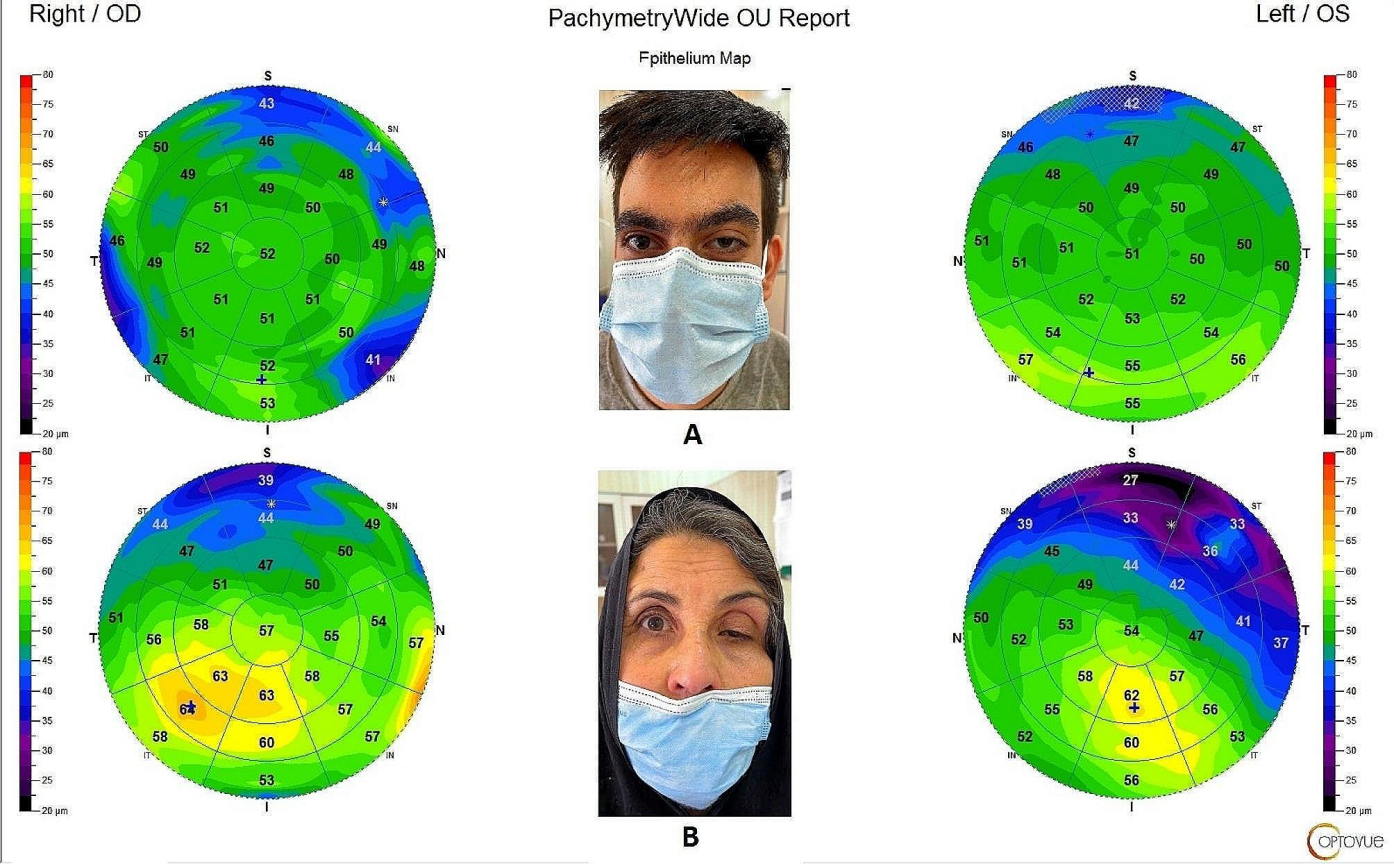
Epithelial thickness map of two patients. **(A)** a young 21 years old man with unilateral left congenital blepharoptosis. **(B)** a middle-age 53 years old woman with unilateral left aponeurotic blepharoptosis. Both figures show thinner superior corneal epithelial thicknesses in ptotic eye in comparison with contralateral normal eye

During all scans, one educated assistant was instructed to lift the ptotic eyelid very gently without any pressure to the eyeball. After the exam, image quality of each image was evaluated and images with poor quality were discarded. Only images with good quality (image quality score more than 30 was considered as good quality) were included for analysis (Fig. 1).

### Statistical analysis

Post hoc power: As the main outcome of this study was the difference in corneal epithelial thickness between ptotic and contralateral non-ptotic eyes in different areas of the cornea, we calculated the maximum required sample size for all these parameters. This was based on the maximum observed standard deviation of the parameters in each group (4.89 in the ptotic group and 4.69 in the contralateral non-ptotic group) and for at least a 10% difference in the parameter from the ptotic eye (here, the minimum difference has been considered to maximize the sample size). So, the maximum required sample size with a power of 95% was calculated to be 27. We had 44 samples in this study, which exceeded the observed power by more than 95% for the mentioned variables.

IBM SPSS Statistics for Windows software (version 25, IBM Corp.) was used for statistical analysis. A p-value of less than 0.05 was considered significant.

We presented data using mean, standard deviation, median, and range. To compare outcomes between two eyes considering the probable correlation of measurements, we used GEE (generalized estimating equation). To assess the relation of variables, we used the Spearman correlation coefficient.

To control inflation of type I error (multiple comparisons have been performed), we report Benjamini & Hochberg’s [32] adjusted p-values in addition to the raw p-value in Table 1.

**Table 1:** Comparison of epithelial thickness between ptotic and contralateral non-ptotic eyes

Epithelial thickness in 6 mm zone (microns)	Ptotic eye	Contralateral non-ptotic eye	*P*-Value	adjusted *p*-value
Temporal sector	51.54 ± 3.71	52.07 ± 3.88	0.174	0.209
Superior-temporal sector	48.92 ± 3.95	50.82 ± 3.64	< 0.001	< 0.001
Superior sector	48.07 ± 4.43	50.40 ± 4.00	< 0.001	< 0.001
Superior-nasal sector	50.53 ± 4.89	51.75 ± 4.21	0.005	0.010
Nasal sector	53.31 ± 4.70	52.94 ± 3.87	0.307	0.335
Inferior-nasal sector	55.01 ± 4.45	54.26 ± 3.64	0.029	0.050
Inferior sector	55.30 ± 4.34	54.77 ± 4.69	0.104	0.139
Inferior-temporal sector	53.72 ± 4.47	53.77 ± 3.81	0.908	0.908
Minimum thickness	47.81 ± 4.37	49.50 ± 3.92	< 0.001	< 0.001
Maximum thickness	56.66 ± 4.67	56.05 ± 4.61	0.042	0.063
Min–Max thickness	-8.86 ± 3.58	-6.55 ± 3.11	< 0.001	< 0.001
Standard deviation	2.25 ± 1.05	1.59 ± 0.80	< 0.001	< 0.001

## Results

52 cases with unilateral blepharoptosis were initially enrolled. Eight cases were excluded because of history of refractive surgery (2 cases), contact lens use (3 cases), dry eye (1 case) and poor cooperation during examinations (2 cases). Finally, 44 patients with unilateral blepharoptosis were included in the study.

27 (61.4%) of the patients were female and 17 (38.6%) cases were male. The mean age of the patients was 24.40 ± 15.16 years with a range of 6 to 71 years. Regarding the type of blepharoptosis, most cases were congenital (30 (68.2%) patients). Among the rest of cases, 11 (25.0%) were acquired aponeurotic and in 3 (6.8%) cases the clinician was not sure about the exact cause of blepharoptosis (Table 2).

**Table 2:** Summary of demographic and blepharoptosis related findings of the patients

	Minimum	Maximum	Mean ± SD
Age (years)	6.00	71.00	24.40 ± 15.16
Duration of ptosis (years)	0.66	71.00	16.42 ± 13.63
Ptotic eye MRD1 (mm)	-4.00	3.50	1.06 ± 1.67
Contralateral non-ptotic eye MRD1 (mm)	3.00	6.00	4.05 ± 0.95
Ptotic eye LPS function (mm)	0.00	16.00	8.46 ± 5.02
Contralateral non-ptotic eye LPS function (mm)	10.00	18.00	14.88 ± 2.02

**Fig. 2:**
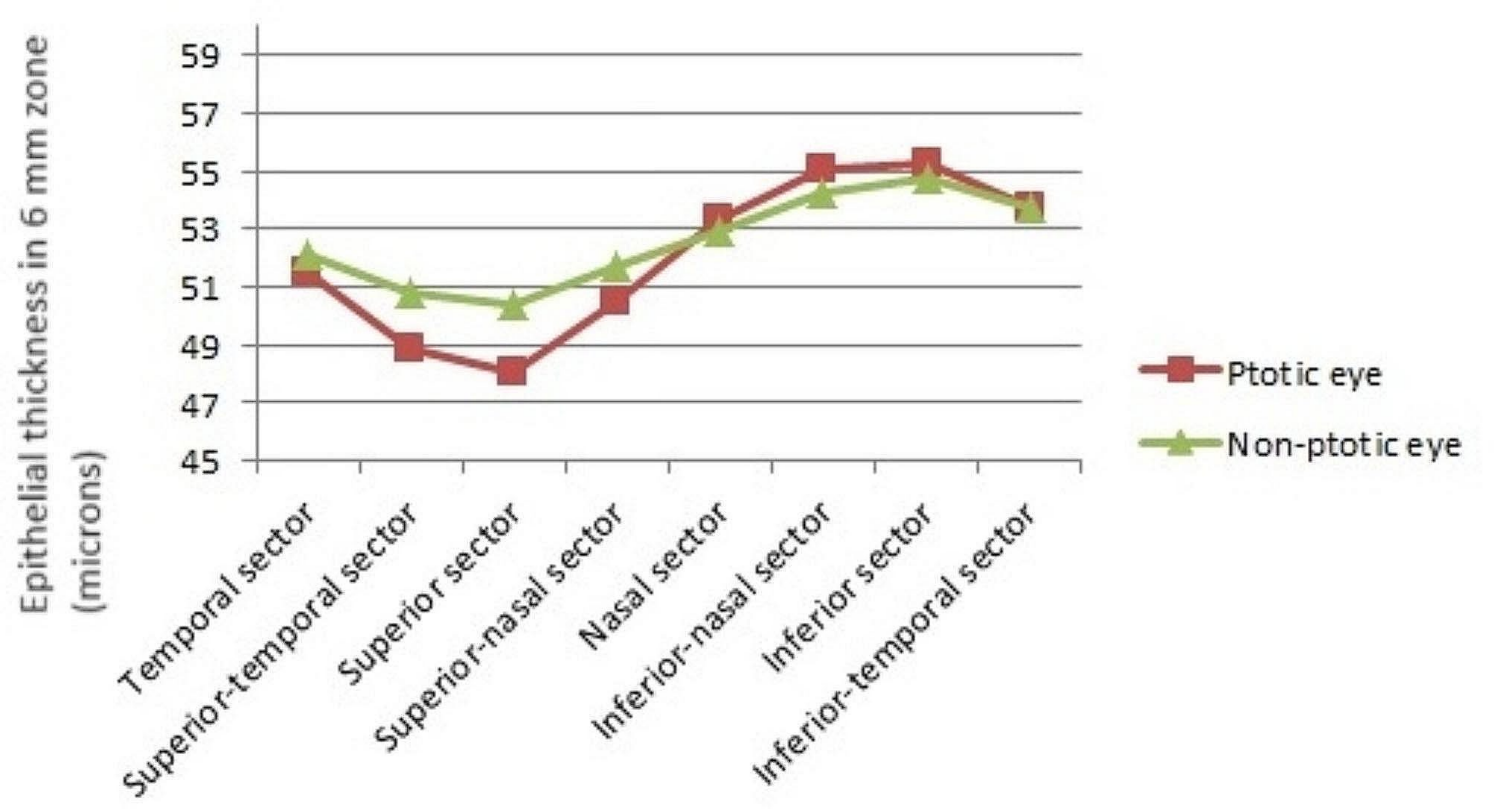
The comparison graph of the corneal epithelial thickness in different corneal sectors. As shown in the figure, the superior sectors of the ptotic eyes have thinner thicknesses

Comparing the 6 mm ETM sectors of normal versus ptotic eyes, ptotic eyes had significantly thinner superior, superior-temporal and superior-nasal sectors of the cornea. On the other hand, no difference was found in the temporal, nasal, inferior and inferior-temporal sectors and slightly thicker CE in the inferior-nasal sector (Table 1; Fig. 2).

In another analysis, the correlation of difference of superior-inferior CE was evaluated with different parameters including patient’s age (*p* = 0.457), type of blepharoptosis (*p* = 0.786), duration of blepharoptosis (*p* = 0.477) and MRD1 (*p* = 0.248). But no correlation was found.

There was no significant difference in anterior corneal power between the two groups (*p* = 0.26).

## Discussion

Despite its stability as the first covering layer of the cornea, epithelium has the great ability to alter in different conditions [2, 3]. Several studies were conducted, by means of different imaging modalities, to show the changes or differences of this layer in a variety of ophthalmic situations. In evaluation of normal cornea, Using VHFU technology, Reinstein et al. indicated that the average central CET was 53.4 ± 4.6 μm, comparable with SD-OCT [8]. Using SD-OCT, normal CET profiles were defined to be thicker in the central 2 mm of the cornea ranging from 53 to 54 μm in the center and decreased with a low gradient of -0.43 μm/mm toward 7 mm mid-periphery and a larger gradient of -2.31 μm/mm toward 9 mm periphery [10, 33–37]; but VHF-US studies did not find such thinning trends [8]. As previously mentioned, with rare exceptions [38], almost all studies found CET to be thicker in the inferior cornea than in the superior cornea of normal eyes [6–20]. This difference may range from 3 to 5.7 μm using VHF-US and 2.2 [18] to 4.4 μm [11] using OCT. A similar difference is also noted in children of different ethnic groups [10, 39, 40] and was measured by one study as 3.3, 3.5, and 3.6 μm in the paracentral, midperipheral, and peripheral areas, respectively [10]. Some authors attributed this difference to mechanical rubbing of the upper eyelid to superior corneal epithelium, possibly thinning the superior epithelial thickness [18, 21, 22]. In this study, this superior-inferior difference was noted again.

Although many authors were interested in the effect of aging on the CET profile, this topic remains one of the most debated ones in the literature. While some authors found negligible differences in the majority of zones of the CET profile of adults as a result of aging [8, 12, 14, 22, 25], others found a steady decrease in CET in the older age groups [16, 41]. However, total CET profiles may show higher variability in older age groups [12, 23, 24], particularly in the superior parts of the cornea [25]. These changes could be either due to the structural changes of the cornea during the process of aging or other neighboring parts as eyelid which could have pressure effects particularly on the superior parts of the cornea. But in this study no correlation was found between difference of superior-inferior CE and age.

Physiological fluctuations in corneal thickness and curvature were reported during the day and night. These fluctuations were reported to be about 19 and 22 μm in the central and peripheral cornea, respectively [38]. During sleep time, relative hypoxia and increased temperature under closed eyelids may happen. These factors in combination with reduced uveoscleral outflow and increased IOP, may cause a corneal swelling [42, 43]. So we tried to eliminate the diurnal confounding factors in this study.

There is a growing body of evidence in the field of ETM changes in conditions with contact with the epithelial surface. In this regard, the effect of the eyelid abnormalities and disorders or hard and soft contact lens use has been evaluated in several studies.

Regarding the use of contact lenses, short term (eight hours) wearing of daily disposable soft contact lenses (SCL) of different brands may not result in significant ETM alterations [44] but it has been shown that long-term usage of SCL can cause significant thinning of CET profile in all central, paracentral, and midperipheral sectors [45, 46].

Comparison of ETM in thirty eyes with allergic conjunctivitis with their normal counterparts using OCT showed a significant decrease in average CET of paracentral (2–5 mm) and midperipheral (5–7 mm) annuli, with a negative correlation with eye rubbing frequency and allergic signs severity and also a higher ETM SD in allergic patients [28].

In a study by Arslan et al., the effect of dermatochalasis and upper eyelid blepharoplasty (UEB) on CET changes was evaluated using AS-OCT. 90 eyes with dermatochalasis were divided into 3 groups (according to the severity of the dermatochalasis) and were compared with 41 control eyes. Their study showed statistically significant differences in the CET of the superior, superonasal, superotemporal, inferotemporal, and temporal sectors between the dermatochalasis and control groups (*p* < 0.05). Their results also showed no differences in CET among the sectors in the control group, while there was a significant difference in CET among some sectors of the dermatochalasis group. There was no significant difference in CET between the severity subgroups across all sectors both preoperatively and postoperatively. In 6-month follow-up measurements after UEB surgery, the CET in all sectors increased statistically significantly compared to preoperative period [47].

In another study, ETM of 13 eyes with mild congenital myogenic ptosis (< 2 mm) was compared with 13 normal eyes. The study reported significantly thinner superior ET and higher ETM SD, yet both groups had similar total ET. The authors attributed these changes to the thinning of the superior ET in all sectors in the ptosis group compared to inferior sectors [29]. Our study was the first one in which the ptotic eyes of unilateral blepharoptosis patients was compared with the contralateral non-ptotic eye. In our study, a similar difference between superior and inferior parts of the corneal epithelium was shown.

Focal thinning in the central CE due to a large central chalazion of the upper eyelid has been reported; it resolved completely after successful treatment of the lesion [48].

In fact, comparison of ETM in normal individuals has a large variation [49]. In this regard, we decided to evaluate the ETM in blepharoptosis patients in a contralateral eye study for the first time in the literature as far as we know. ETM of normal individuals were shown to be comparable in both eyes in several studies. As a result, we eliminated the effects of daytime, age, sex or other factors in the literature and diseases that have known effects on the ETM. In our study, we found a significant decrease in the superior, superior-temporal, superior-nasal and central sectors of the ETM while no difference in temporal, nasal, inferior and inferior-temporal sectors were found. On the other hand, we found a thicker inferior- nasal ETM in the ptotic eye. This is in the line with the theory of thinning effect of the eyelid on the ETM. Furthermore, there were higher variability (min-max) and standard deviation in the ptotic eyes which was the same finding as of the Dogan et al. study [29]. We show that, surprisingly, there was no positive correlation between the MRD1, type and duration of blepharoptosis and the difference of superior and inferior epithelial thickness. The limitations of this study included of small sample size, gathering a group of both congenital and aponeurotic in both children and adult groups of patients.

In conclusion, our study demonstrates a significant association between blepharoptosis and altered corneal epithelial thickness, particularly in the superior corneal sectors. A thinning effect on superior corneal sectors may happen because of the lower position of the upper eyelid in blepharoptosis. These changes in the superior area of the cornea can be due to the effect of mechanical rubbing of the eyelids for a long time. A healthy corneal epithelium is crucial for maintaining tear film stability and ocular surface integrity. Changes in the thickness of this epithelium can reduce corneal health and cause potential visual problems. Abnormal epithelial thickness can be a marker for dry eye disease, a common complication associated with post-surgical ptosis repair. Understanding this link might be valuable in diagnosing and guiding treatment decisions for these patients. Additionally, monitoring changes in epithelial thickness could be used to assess the effectiveness of ptosis repair surgeries in preserving corneal health. Future studies are warranted to investigate the mechanisms underlying these changes and explore the potential clinical applications of our findings.

## Data Availability

The datasets used and/or analysed during the current study are available from the corresponding author on reasonable request.
